# Bovine viral diarrhea virus in free-ranging wild ruminants in Switzerland: low prevalence of infection despite regular interactions with domestic livestock

**DOI:** 10.1186/1746-6148-8-204

**Published:** 2012-10-29

**Authors:** Julien Casaubon, Hans-Rudolf Vogt, Hanspeter Stalder, Corinne Hug, Marie-Pierre Ryser-Degiorgis

**Affiliations:** 1 Centre for Fish and Wildlife Health (FIWI), Vetsuisse Faculty, University of Bern, Bern, Switzerland; 2 Institute of Veterinary Virology (IVV), Vetsuisse Faculty, University of Bern, Bern, Switzerland

**Keywords:** Bovine viral diarrhea virus, Epidemiology, Interactions, Livestock, Seroprevalence, Switzerland, Wild ruminants

## Abstract

**Background:**

In the frame of an eradication program for bovine viral diarrhea (BVD) in Swiss livestock, the question was raised whether free-ranging wildlife could threaten the success of this sanitary measure. Therefore, we conducted serological and virological investigations on BVD virus (BVDV) infections in the four indigenous wild ruminant species (roe deer, red deer, Alpine chamois and Alpine ibex) from 2009 to 2011, and gathered information on interactions between wild and domestic ruminants in an alpine environment by questionnaire survey.

**Results:**

Thirty-two sera out of 1’877 (1.7%, 95% confidence interval [CI] 1.2-2.4) were seropositive for BVDV, and a BVDV1 sub genotype h virus was found in a seropositive chamois (0.05%, 95% CI 0.001-0.3). The seropositive animals originated from sub-alpine or alpine regions and significantly more seropositive red deer, chamois and ibex than roe deer were found. There were no statistically significant differences between sampling units, age classes, genders, and sampling years. The obtained prevalences were significantly lower than those documented in livestock, and most positive wild ruminants were found in proximity of domestic outbreaks. Additionally, BVDV seroprevalence in ibex was significantly lower than previously reported from Switzerland. The survey on interspecific interactions revealed that interactions expected to allow BVDV transmission, from physical contacts to non-simultaneous use of the same areas, regularly occur on pastures among all investigated ruminant species. Interactions involving cervids were more often observed with cattle than with small ruminants, chamois were observed with all three domestic species, and ibex interacted mostly with small ruminants. Interactions related to the use of anthropogenic food sources were frequently observed, especially between red deer and cattle in wintertime.

**Conclusions:**

To our knowledge, this is the first report of BVDV RNA isolated from an Alpine chamois. Nevertheless, our results suggest that BVDV infections are only sporadic in Swiss wild ruminants, despite regular occurrence of interactions with potentially infected livestock. Overall, serological, virological and ethological data indicate that wildlife is currently an incidental spill-over host and not a reservoir for BVDV in Switzerland.

## Background

Environmental and socio-economical changes have lead to an increase of interactions between wild and domestic species worldwide, a phenomenon that has received a growing attention during the past decade. It is now largely recognized that wildlife can play important roles in the epidemiology of infectious diseases shared between wild and domestic species [1]. In particular, the potential of wild animals as pathogen reservoirs and sources of infection for domestic livestock has been of increasing concern [2]. Thus, when an eradication program was implemented for bovine viral diarrhea (BVD) in domestic livestock in Switzerland, the question was raised whether free-ranging wild ruminants - roe deer (*Capreolus c*. *capreolus*), red deer (*Cervus e*. *elaphus*), Alpine chamois (*Rupicapra r*. *rupicara*) and Alpine ibex (*Capra i*. *ibex*) - may represent a threat to the success of the program.

Bovine viral diarrhea (BVD) is responsible for massive economic losses [3] in livestock farming. It is caused by the BVD virus (BVDV), an RNA-virus that belongs to the genus Pestivirus of the family *Flaviviridae*. This virus family also comprises the pathogens causing classical swine fever and border disease. BVDV is transmitted mainly horizontally from animal to animal via oral and nasopharyngeal secretions, or iatrogenically (rectal examinations, injections with contaminated needles, contaminated live vaccines) [4]. An acute infection usually remains inapparent or causes only mild disease and establishes a robust immunity. The importance of BVDV is due to the occurrence of persistently infected (PI) animals, resulting from infections of pregnant cows or heifers during gestation prior to the development of the fetus’ immune system. In such a case, the virus is recognized as “self”, and an animal born alive will spread BVDV lifelong. Persistently infected cattle may develop the fatal mucosal disease [5]. The isolation of BVDV and the occurrence of PI animals have been documented in various free-ranging and captive species [6,7] but the possible role of wild ruminants as reservoir remains unclear.

Previously to the national BVD eradication program, started in 2008 with the aim of eliminating all PI animals [8], BVD was endemic in the Swiss cattle population with a seroprevalence of 57.6% [9]. Subsequently, the prevalence of PI cattle dropped from 1.8% to 0.2% in the first phase of the program [10]. Several studies on Pestivirus infections in red deer, chamois and ibex, either antibody or antigen surveys, have been conducted in Switzerland in the past; however all of them were limited in some way, considering only one animal species and/or a single specific geographical region [11-13].

Interactions between wild and domestic ruminants are not rare in Switzerland [14,15] and it is likely that their occurrence increased during the second half of the past century because of the rise of indigenous Swiss wild ruminants during this period and of the implementation of an agricultural production based on high productivity. The latter has lead to new and highly productive pasturing surfaces from which the wild populations have benefited [16]. The occurrence of interactions between wild and domestic species has also been documented in other countries [17,18] and more attention has been directed toward the potential role of wildlife in the epidemiology of diseases that may be shared between wild and domestic species [19]. However, while the occurrence of such interactions is often reported in scientific articles, the information remains vague or not properly documented and there is a need for more systematically collected data.

The aims of the present study were to assess the potential role of free-ranging wild ruminant populations as a source of (re-)infection with BVDV for domestic livestock, and to quantify and characterize interactions among domestic and free-ranging ruminant species. Therefore, 1) we carried out a cross-sectional study to determine the current apparent prevalence of BVDV infections in the four species of free-ranging indigenous wild ruminants in Switzerland in parallel to the BVD eradication program in livestock, and compared our results with previous studies; and 2) we performed a questionnaire survey to document the occurrence and type of interactions between domestic and wild ruminant species in an alpine environment.

## Results

### Bovine viral diarrhea virus

**Figure 1:**
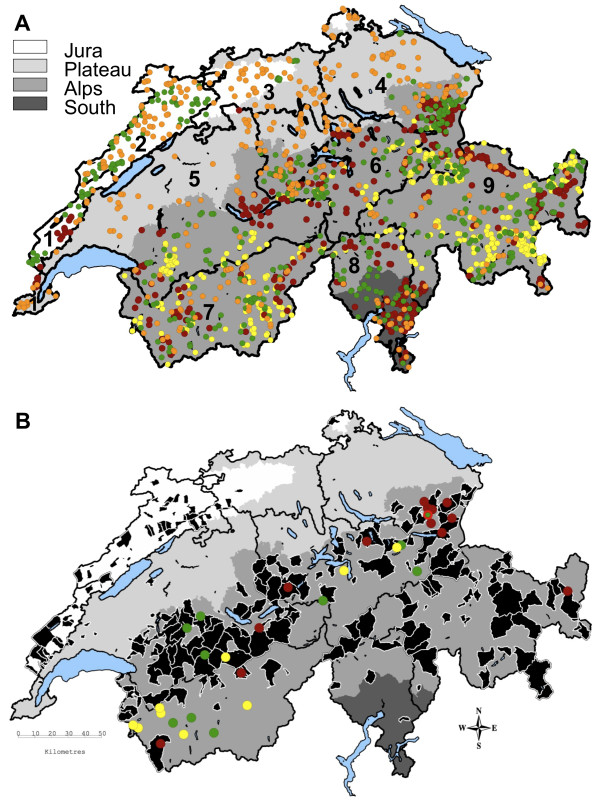
**Map of Switzerland showing (A) sampling units and sampled animals and (B) seropositive animals.** Grey shaded areas represent different bioregions, major lakes are in blue. Numbers refer to sampling units: 1) Jura-South, 2) Jura-North, 3) North-West, 4) North-East, 5) Centre-West, 6) Centre-East, 7) South-West, 8) South-Centre, 9) South-East. Colored dots correspond to animals of different species: Orange: roe deer; Red: red deer; Green: chamois; Yellow: ibex. The seropositive chamois (green dot in Figure 1B) located in the unit North-East and indicated by a bright red halo was also positive by PCR. Black areas in Figure 1B refer to communities in which at least one domestic animal was found positive for bovine viral diarrhea virus (persistently infected cattle detected during the summer pasturing season in the frame of the eradication program; data obtained from the Swiss Federal Veterinary Office).

Serum samples from 1’877 wild ruminants (435 roe deer, 476 red deer, 466 chamois, 500 ibex; Figure 1A) were screened by ELISA for BVDV antibodies. In this first screening, 77 samples showed a positive or indeterminate reaction. In the subsequent serum neutralization test (SNT), 32 of the 77 samples (13 red deer, 10 chamois, nine ibex; Figure 1B) showed antibody titers ranging from 1:8 to 1:861. Overall, apparent seroprevalence was 1.7% (95% confidence interval [CI] 1.2-2.4) (Table 1). The seropositive animals originated from sub-alpine or alpine regions (Figure 1B) and significantly more seropositive red deer (*p* < 0.001), chamois (*p* < 0.001) and ibex (*p* = 0.004) than roe deer were found. There were no statistically significant differences between sampling units, age classes, genders, and sampling years.

**Table 1:** Detection of antibodies against BVDV in four species of wild ruminants in Switzerland

		**Sampling units**									**Total**
		**Jura**	**Jura**	**North**	**North**	**Centre**	**Centre**	**South**	**South**	**South**	
		**South**	**North**	**West**	**East**	**West**	**East**	**West**	**Centre**	**East**	
Roe deer	Seroprevalence	0	0	0	0	0	0	0	0	0	0
	(95% CI)	(0–14.8)	(0–5.4)	(0–6)	(0–4.5)	(0–9)	(0–5)	(0–8)	(0–21.8)	(0–10)	(0–0.8)
	positive/tested	0/23	0/67	0/60	0/80	0/39	0/72	0/44	0/15	0/35	0/435
Red deer	Seroprevalence	0	-	-	6.9	1.8	4.8	3.6	0	0.8	2.7
	(95% CI)	(0–20.6)			(2.8–13.8)	(0.05–9.5)	(0.6–16.2)	(0.4–12.5)	(0–4.6)	(0.02–4.3)	(1.5–4.6)
	positive/tested	0/16	-	-	7/101	1/56	2/42	2/55	0/78	1/128	13/476
Chamois	Seroprevalence	0	0	0	1.4	11.5	4.8	5.6	0	0	2.1
	(95% CI)	(0–13.2)	(0–5.8)	(0–36.9)	(0–7.5)	(2.4–30.2)	(1.0–13.3)	(1.2–15.4)	(0–9.5)	(0–3.1)	(1.0–3.9)
	positive/tested	0/26	0/62	0/8	1/72	3/26	3/63	3/54	0/37	0/118	10/466
Ibex	Seroprevalence	-	-	-	0	1.2	4.3	6.7	0	0	1.8
	(95% CI)				(0–6.3)	(0.03–6.7)	(0.5–14.5)	(2.5–14.1)	(0–33.6)	(0–1.7)	(0.8–3.4)
	positive/tested	-	-	-	0/57	1/81	2/47	6/89	0/9	0/217	9/500
Total	Seroprevalence	0	0	0	2.9	2.5	3.1	5	0	0.2	**1**.**7**
	(95% CI)	(0–5.5)	(0–2.8)	(0–5.3)	(1.3–5.4)	(0.8–5.7)	(1.3–6.3)	(2.6–8.5)	(0–2.6)	(0.01–1.1)	(1.2–2.4)
	positive/tested	0/65	0/129	0/68	9/310	5/202	7/224	12/242	0/139	1/498	32/1877

**Figure 2:**
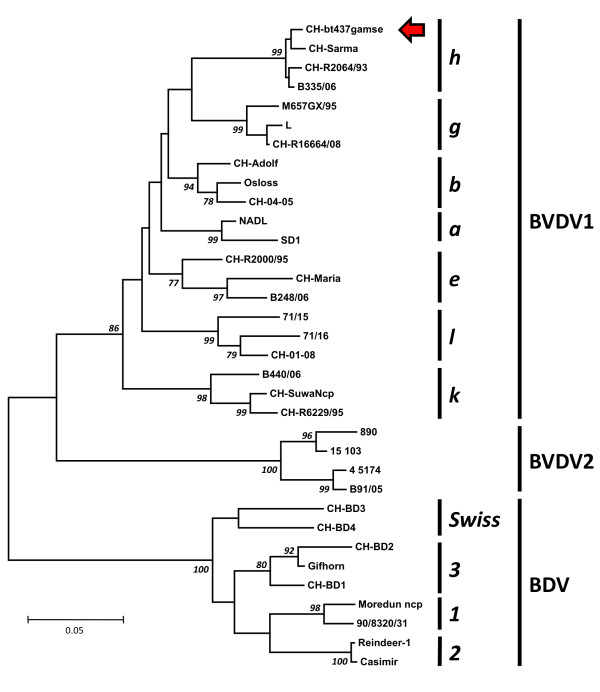
**Unrooted phylogenetic tree based on the 5’UTR (229 positions) of bovine viral diarrhea virus (BVDV).** The chamois virus sequence (red arrow) is shown in the context of other pestiviral isolates from cattle and small ruminants in Switzerland to date. Swiss isolates are labeled with CH- preceding the name. Additional sequences were obtained from GenBank. BVDV1 subgenotype a, b, e, g, h, k and l, BVDV2 and BDV 1, 2, 3 and Swiss are shown. From BVDV1 subgenotype g and l, only one isolate each has been found in Switzerland, whereas subgenotypes h, e, k and b are common. The BDV Swiss genotype has been found only in Switzerland so far. Branch numbers (italics) indicate the percentage of 2000 bootstrap replicates [4]. Only bootstrap values over 75 are shown. Branch lengths are proportional to genetic distances, and the bar shown indicates 0.05 substitutions per site.

Viral RNA was detected by real-time RT-PCR in one seropositive chamois kid (Figure 2B), and results were not interpretable for eight further seropositive animals (no amplification of the internal control). All other samples (seronegative or seropositive) were PCR-negative. Thus, the estimated viral prevalence was 0.05% (1/1’836, 95% CI 0.001-0.3). The RNA sequence from the chamois kid sample could be assigned to the previously described BVDV1 sub genotype h [5] (Figure 2). 

Comparing our results with the situation in domestic livestock (data from the Swiss Federal Veterinary Office), almost all positive wild ruminants were sampled within or at the periphery of a community with at least one PI cattle detected during the summer pasturing season in the frame of the eradication program (Figure 1B). However, no wild animals from the Jura were positive despite the presence of PI cattle. Seroprevalence in cattle prior to the eradication program (57.6% [9]) was significantly higher (*p* < 0.001) than the 1.7% that we report for wildlife. Virus prevalence in Swiss cattle born prior to 2008 (0.81% [10]), i.e., before the start of the eradication program, was also significantly higher (*p* < 0.001) than the 0.05% obtained in wild ruminants in our study. The sequence obtained from the PCR product [GenBank: JX297593] was identical with sequences from cattle strains (100% match in the 5’UTR region) detected from eight different cattle during the eradication program. Three of these cattle were born within a radius of 10 km around the sampling location of the chamois.

### Interspecific interactions

The majority of the contacted game wardens participated to the questionnaire survey (67% response rate for the first questionnaire and 83% response rate for the second one). Roe deer, red deer, chamois and cattle were reported to be present in all sub-districts while ibex, goat and sheep occur in only part of them. The period of observation ranged from 0.5 to 41 years with a mean of 17 years. Detailed data on the answers can be found in the Additional files 1, 2, 3, 4, 5, 6 and 7. Hereafter, rare observations are not taken into account, i.e., statistical analyses focus on observations made more than once a year.

**Figure 3:**
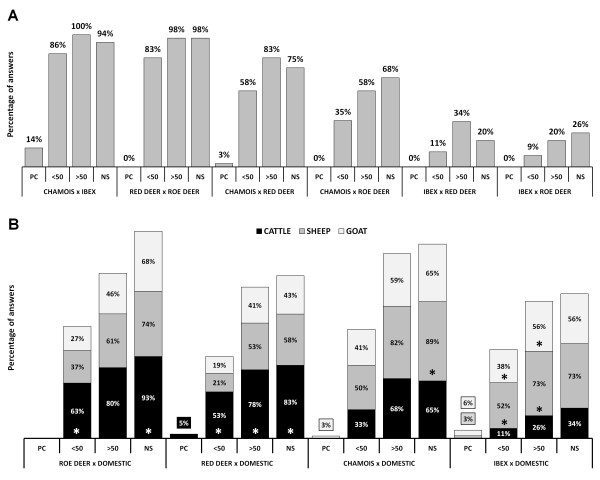
**Answers to the questionnaire survey on interspecific interactions in Graubünden, Switzerland: proximity between species.** (**A**) Interactions among wild ruminants; (**B**) Interactions between wild and domestic ruminants. PC: physical contact; < 50: encounter of < 50 m; > 50: encounter of > 50 m; NS: non-simultaneous occupation of the same area. Asterisks indicate statistically significant difference (Fisher’s Exact Test, *p* < 0.05) from the other group(s).

Data on proximity between species are summarized in Figure 3. Considering wild species only, interspecific interactions are observed mainly within the same taxonomic family, i.e., among caprids (chamois and ibex) and among cervids (roe deer and red deer), but more than half of the game wardens also reported encounters between chamois and cervids, including rare physical contacts between chamois and red deer. In contrast, interactions between ibex and cervids were reported by only one third of the game wardens and no physical contact was observed.

**Figure 4:**
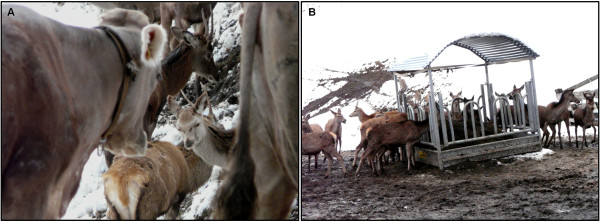
**Interactions between red deer and cattle in Graubünden, Switzerland.** (**A**) Encounter of < 50 m between cattle and red deer. (**B**) Red deer aggregation in relationship with anthropogenic food resources, here the use of hay silage for livestock in winter. Courtesy of Martin Michael.

Regarding wildlife and domestic livestock, encounters of < 50 m and non-simultaneous occupation of the same area were documented significantly more frequently between roe deer and cattle than with sheep or goat. For red deer, encounters of < 50 m (Figure 4A), encounters of > 50 m and non-simultaneously occupation of the same area were observed most frequently with cattle, and 5% of the game wardens reported physical contact between the two species. For chamois, only non-simultaneous occupation of the same area was significantly more observed with sheep than with the other species. One game warden reported physical contact between chamois and goat. For ibex, encounters of < 50 m and > 50 m were observed more frequently with domestic small ruminants than with cattle, and physical contact was reported with both sheep and goat.

Regarding the duration of encounters, the wild species most often observed in contact together for > 1 h were chamois and ibex. Sixty-six percent of the game wardens regularly observed such encounters. Nevertheless, 33% of the game wardens reported long-lasting encounters between chamois and red deer, 30% between roe deer and red deer, 18% between chamois and roe deer, and 6% between ibex and red deer.

The duration of encounters between wild and domestic species shows the same frequency pattern as for the proximity, i.e., interactions of > 1 h involving cervids were more often observed with cattle than with small ruminants (*p* = 0.006 to *p* = 0.029), chamois were observed with all three domestic species (in similar proportions), and ibex interacted for long intervals only with small ruminants (*p* < 0.021).

All four types of situations were observed between chamois and ibex (mixing of herds when grazing: 80%, use of the same natural feeding resource: 94%, use of the same salt lick: 86% and use of the same resting places: 71%). The observations were less frequently reported between roe deer and red deer (mixing of herds when grazing: 50%, use of the same natural feeding resource: 78%, use of the same salt lick: 48%, use of the same resting places: 50%). Chamois were observed more frequently sharing the same salt lick (*p* = 0.039) and resting places (*p* = 0.01) with red deer than with roe deer, and ibex were observed more frequently sharing the same natural feeding resource (*p* = 0.001) and salt lick (*p* = 0.011) with red deer than with roe deer.

Interactions between roe deer and domestic ruminants such as the use of the same natural feeding resource and mixing of herds when grazing, were observed more frequently with cattle than with sheep and goats (*p* < 0.034). All types of interactions involving red deer were more frequently observed with cattle than other domestic species but the difference was statistically significant only for the use of the same natural feeding resource (*p* < 0.021). For chamois, there were no significant differences regarding the frequency of observations with domestic ruminants but all four situations were observed slightly more often with sheep than with goat and cattle. For ibex, three out of four types of interactions (use of the same natural feeding resource, of the same salt lick and of the same resting places) were more often observed with sheep plus goat than with cattle (*p* < 0.005).

Anthropogenic food resources were reported to be frequently used by wild ruminants. A common use of the same wildlife feeding places was observed between roe deer and red deer (18% of the game wardens) and rarely between red deer and chamois (2%) or roe deer and chamois (2%). Food sources meant for domestic livestock are reported to be used in majority by roe deer and red deer (Figure 4B) and in relationship with the three domestic housing facilities (cattle, sheep, goat) but significantly more often in relationship with cattle housing (22% for roe deer, 54% for red deer) than with sheep or goat housing (*p* < 0.05). Interspecific interactions among cervids were observed at such places by 32% of the game wardens. Cervid interactions driven by other food sources (use of garden waste or fallen fruits, intentional feeding with e.g. hay) on private ground are even more common (46%).

General comments made by game wardens were particularly abundant on the topic of interactions related to anthropogenic food resources. All these interactions were observed exclusively during winter and early spring. Intentional feeding apparently occurs mostly above the upper forest line, i.e., at 1’800 to 2’000 m above sea level (a.s.l.) where cut grass of poor quality is made accessible for wild species in winter times. Wild species were reported to use livestock food mostly at night, when livestock is not around. The most commonly used anthropogenic food source is silage (in form of silage bales) especially in relationship with outdoor housing of livestock (run-outs), i.e., wild species tend to enter livestock enclosures. If unprotected, silage reserves are easily accessible and attract many wild animals (20–30 individuals at the same place). Most farms known to be regularly visited by wild ruminants are located at the bottom of Alpine valleys (between 500 and 1’500 m a.s.l.) and are therefore accessible mainly only for cervids. Other food sources on private grounds (composting kitchen waste, fallen fruits, bread intentionally spread for deer) are found in nearly each community and have also an attractive effect on wildlife, especially in winter.

## Discussion

In this study, we assessed for the first time the prevalence of BVDV infections in all four indigenous wild ruminant species on the whole territory of Switzerland. Results provide a general picture of the current BVDV infection status of these species, which is necessary for the future planning and successful achievement of the implemented BVD eradication program in livestock. Although the number of investigated animals was too low to provide strong data at local level, the total sample size (> 400 animals tested per species) fulfilled criteria both for detection of “disease” (virus positive animals) and prevalence estimation at country level.

This study also provides valuable information on the occurrence of interspecific interactions among wild ruminants and between wild and domestic ruminants in an alpine environment. These quantitative data are necessary to understand the epidemiology of infectious diseases in natural habitats used by different animal species and to perform risk assessments. Data gathered by means of questionnaire surveys rely on observations of a number of voluntary participants and may be biased in various ways. Furthermore, exact and detailed information is nearly impossible to collect because 1) participants have to rely on memorized events to answer the questions, and 2) the response rate is expected to be negatively influenced by extensive question lists. However, this method represents a relatively easy and efficient way to obtain general information on a large geographical area over a long period of time.

The BVDV screening test gave more positive results than the confirmation test: only 42% percent (32/77) of the samples that were found positive by ELISA were confirmed as such by SNT. Two factors may account for this difference: First, it may be due to poor serum quality and cytotoxicity, which limits performance of the SNT and hampers result interpretation. Results that could not be validated by SNT were classified as non-interpretable, which may have lead to an underestimation of the final seroprevalence. Second, cross-reactions with other potential circulating pestivirus may have occurred in the ELISA. Because only one virus strain was used for the SNT, it was not possible to assess the occurrence of such cross-reactions [20]. Other studies have indeed shown a high circulation of pestivirus in wild ungulates populations, e.g. the Border disease virus (BDV) in ibex and chamois in the Italian Alps [21,22] or in chamois in France and Spain [23-25], and it is likely that this virus also circulates in Swiss wildlife. The presence of other pestivirus than BVDV could also explain the presence of seropositive wild animals in communities without documented PI cattle (e.g. unit South-West).

Overall, we observed low seroprevalences in red deer, chamois and ibex and no antibodies were detected in roe deer. For red deer, the seroprevalence calculated in the present study (2.7%) is higher than previously reported in Switzerland (1.7%, 95% CI 0.46-4.25 [11]) but the difference is not significant (*p* = 0.602). Our results are in line with a study from Austria (2% [26]), a country that started an eradication program for BVDV in livestock at regional level in 1997 and at national level in 2004 [27]. Other European countries have reported low seroprevalences (0% to 5%) in red deer populations [28-33].

Similarly, the situation in Swiss chamois is comparable to previous studies in Europe, which documented BVDV seroprevalences ranging from 3.4% to 5.5% [28,31,34]. In ibex, seroprevalence has significantly decreased [12], both at local level (from 3.9% to 0% in the unit South-East, *p* = 0.003) and at national level (from 4.3% to 1.8%, *p* = 0.021). In the unit South-West, a non-significant decrease was also noted (from 8.8% to 6.7%, *p* = 0.613). If this decline was a consequence of the implementation of the BVD eradication program in 2008, the prevalence is expected to continue to decrease in wild populations.

Concerning roe deer, previous reports have documented low seroprevalences in Germany (0% to 0.7% [28,35,36]) and in France (0.7% [34]). However, in Germany higher prevalences reaching up to 9.8% have also been recorded, pointing at existing regional differences [31-33]. A distinct BVDV-like strain was found in free-ranging roe deer in Germany, indicating that specific BVDV strains might circulate in this species [37]. In Scandinavia, antibodies against BVDV were detected in 12.3% of 635 Norwegian roe deer and the authors also concluded that this situation might be due to the circulation of a wild BVDV-like strain [29].

We report here the detection of bovine-related viral RNA in a seropositive chamois kid. Given the fact that cattle harbouring the same strain were born in the region where this chamois was sampled, and according to the widespread circulation of virus in the cattle population prior to the eradication program, a cattle to chamois transmission is more likely than the contrary. To our knowledge, this is the first report of a detection of BVDV in a chamois and in an Alpine ungulate. There are two possible explanations for the simultaneous presence of virus and antibodies in this chamois kid. On the one hand, it may have had circulating maternal antibodies like it has been described in cattle calves [38]. However, the antibody titer in this chamois (1:861) was much higher than the maternal antibody titer predicted for 3 month-old calves (1:32) [39]. The chamois was > 3 months old and we expect that transfer and persistence of maternal antibodies is comparable in wild and domestic ruminants. On the other hand, it may have undergone an acute infection but the simultaneous occurrence of detectable antibodies and virus in a transiently infected animal is unlikely [40]. To distinguish between a PI animal and a transient infection, two consecutive analyses are necessary, a procedure that is not possible when examining hunted animals.

The facts that all BVDV seropositive animals were located within the Alps, and that the only virus positive wild ruminant was infected with a cattle strain, suggest a role of interactions on alpine pastures as a risk factor for BVDV infection, as already described for cattle infections [41]. Domestic PI animals may have been the source of the infections detected in wildlife.

It is now widely recognized that the epidemiology of diseases is often characterized by multiple host systems [42], and interspecific interactions, especially between wild and domestic species, have received an increasing attention during the past decade. However, although the epidemiological significance of interactions at the wildlife-livestock interface is often incriminated or suspected (e.g. [11,25,43]), such interactions are poorly documented in the internationally available scientific literature. Published studies on interactions between different animal species mostly focus on interferences or influences at the population level - such as mutualism, commensalism, competition and predation - while reports on behavioral and social interactions at the individual level, which are most relevant when talking about pathogen transmission, are scarce [44]. Furthermore, most studies on behavioral interactions between sympatric species focus on only few species, which is likely insufficient when addressing epidemiological questions in a natural environment.

Our data on frequency of interactions originate from a retrospective questionnaire survey and do therefore not present the accuracy of a prospective field study carried out by ethologists. However, they arise from a particularly long observation period (mean of 17 years per district) that also reflects the experience level of the participating game wardens, two factors that are expected to positively influence result reliability. Because the concerned wild species are most active at dawn or dusk [45-48], it is likely that only a small part of the occurring interactions were observed, i.e., their reported occurrence is probably underestimated.

Compared to former questionnaire surveys and prospective observational studies on interactions between domestic sheep, ibex and chamois in the Swiss Alps [14,15,49], we report as many or more observations of 1) physical contacts and short distance encounters between chamois and ibex, and between wild caprids and sheep; 2) mixed herds of chamois and ibex; and 3) encounters at salt licks between chamois and ibex. This supports the expected reliability of our questionnaire data and also shows that rarely observed events like physical contacts are more likely to be recorded by questionnaires than by direct observations on selected study sites. Reports on interactions between domestic goats and wild ruminants had not yet been documented, except for the occasional occurrence of hybrids between goats and ibex [50]. We show here that these interactions are not rare, particularly between goat and ibex.

Interactions among deer species (encounters at < 50 m between roe and fallow deer) were reported to be frequent in an Italian Mediterranean national park [51]. Here we also report frequent encounters of < 50 m and other types of interactions between roe deer and red deer. Interactions between red deer and ibex have already been observed before in the Swiss Alps [52]. However, we report for the first time observations of physical contacts between cervids (red deer) and caprids (chamois). Our data suggest that red deer interact more often with chamois than ibex, which can be explained by the larger overlap of deer and chamois habitat. Similarly, direct interactions between red deer and cattle have already been documented in the Alps [17] but we newly report physical contacts between these two species. Also, we show that roe deer can get close to domestic species, especially cattle, with which almost all types of interactions were reported significantly more often than with small ruminant species. Since the questions on proximity and type of observed interactions were not linked with each other in our questionnaire, it is difficult to conclude from the obtained data which circumstances more likely favor close interspecific contacts. However, additional comments of the participating game wardens and common sense suggest that the simultaneous use of common food sources bear the highest risk for close interactions including physical contacts.

Wildlife feeding has never been performed by the hunting authorities in the canton of Graubünden and was officially abolished 25 years ago by the cantonal hunters’ association (G. Brosi, pers. comm.). However, a number of game wardens reported that intentional feeding of wildlife in wintertime is still common practice, and that interspecific interactions occur at feeding sites, especially between roe deer and red deer. Furthermore, both cervid species but especially red deer were observed near cattle farms - outside or inside livestock enclosures - during winter, profiting of livestock food resources like bale silage. In Switzerland, wildlife management is largely defined at cantonal level and official restrictions regarding wildlife feeding in Graubünden may not apply to other cantons. This means that active wildlife feeding may be even more common in other regions. An aggregation of animals of the same or different species either at intentional or non-intentional feeding places is a recognized risk factor for pathogen transmission [53,54] that needs to be taken into consideration in the frame of disease prevention or control programs.

The risk of pathogen transmission between sympatric species depends on the occurrence, frequency and intensity (i.e., proximity and duration) of interactions. Furthermore, two major transmission pathways have to be considered: 1) by direct contact, i.e., physical contact with an infected host or by contact with the latter’s infected discharges, including contaminated aerosols, feed, water or environment (= direct transmission); or 2) by indirect contact via a living or inanimate intermediate vehicle that transmits pathogens between infected and susceptible hosts, such as arthropod vectors (= indirect transmission) [42,55]. For BVDV, both direct and indirect transmissions are possible [4] and we have shown that all kinds of interactions representing a risk for transmission, including physical contact, short distance encounters and use of the same food resources, are commonly observed between wild and domestic ruminants in the Swiss Alps. Overall, we documented a regular occurrence of interspecific interactions allowing virus transmission.

We showed that BVDV infections are significantly less frequent in wildlife than in Swiss cattle and that seropositive wild ruminants were mostly found in areas with PI cattle. Furthermore, the virus positive chamois was infected with a cattle strain, and seroprevalence in wildlife is apparently decreasing now that the eradication program in livestock is ongoing. It has been previously suggested that conditions to maintain certain pathogens are indeed more favorable in domestic livestock than in wild ruminants [56]: Swiss wildlife populations are much smaller than cattle and sheep populations; furthermore, in livestock intraspecific contacts and mixing of herds (for grazing, shows and markets) and movements throughout the country (commercial exchanges, alpine summering) are more frequent and intense than among wild populations, which is expected to favor pathogen maintenance.

All tested wild animals from the bioregion Jura were negative despite the presence of infected domestic livestock on pastures. Interactions between wild and domestic species are also known to occur in this region but may be less frequent than in the Alps due to the lower number of domestic ruminants on summer grazing pastures. However, data from the Alps suggest that spill-over is rare and our sample size does not allow excluding the infection of single individuals in the Jura.

Although interactions between cattle and roe deer are regularly observed, BVDV antibodies were not detected in this wild species. Earlier studies have shown that roe deer are susceptible to BVDV infection [31-33] and that a distinct BVDV strain exists in roe deer populations from Germany [37]. However, due to the high variability of data obtained for this species across Europe, it seems difficult to draw further conclusions on the susceptibility of roe deer to BVDV cattle strains. The frequent interactions between wild and domestic caprids, and the occurrence of BDV in sheep and goats in Switzerland [57], further underline the need to consider the occurrence of infections with other pestiviruses than BVDV in wild ruminants.

## Conclusions

Our aim was to assess the role of wild ruminants in the epidemiology of BVDV infections in domestic livestock in Switzerland. Here we showed that despite regular occurrence of interspecific interactions, infections in wild ruminants seem to be only sporadic, and we found no indication that the virus circulates among wild populations. We therefore conclude that wildlife is currently an incidental spill-over and not a reservoir host for BVDV, and that it does not represent a threat to the eradication program in livestock. Because data rather suggest that infections of wild animals depend on the situation in livestock, eradication in livestock may also improve the epidemiological situation in wildlife. However, records of BVDV seropositive wild animals in areas without documented PI cattle warrants caution in result interpretation. Especially, it is known for other diseases that spill-over can lead to the slow establishment of wildlife reservoirs and subsequent spill-back [2]. In view of these considerations, performing another country-wide cross-sectional study in a few years may contribute to test the hypothesis that domestic ruminants are the source of BVDV infection for wildlife, i.e., that this viral infection is not maintained in free-ranging wild populations.

## Methods

### Study area and species of interest

The study area comprised the whole territory of Switzerland (41,285 km^2^), which is divided in three main bioregions: the Jura, the Plateau (characterized by a low altitude and high human population density), and the Alps (which may be further divided in various numbers of subregions [58] such as the southern part of the canton of Ticino; Figure 1). In the Alps and, to a lesser extent, in the Jura Mountains, transhumance grazing strategies are common practices, with domestic livestock being brought to alpine pastures (these representing nearly 30% of the total farmland [59]) in summer and brought back to lower altitude and often kept indoor in wintertime. Domestic ruminants registered in 2010 in Switzerland included cattle (1.6 Mio.), sheep (434’083) and goat (86’987) [60]. The most important free-ranging wild ruminants are roe deer, red deer, chamois and ibex. All of them are official game species and populations are mainly regulated through hunting.

### Study design and sampling strategy

This study was based on a cross-sectional convenient sampling strategy to estimate the apparent prevalence of infections with BVDV and a questionnaire survey to gather information on the occurrence of contacts between wildlife and domestic livestock.

Switzerland was divided in nine sampling units (Figure 1) based on political borders and criteria for a survey on Bluetongue virus in Swiss wildlife (Casaubon et al. submitted). Sampling was carried out from August 2009 to April 2011, with a majority of samples collected during two sampling periods which took place during the main hunting period (September-December) in 2009 and 2010. The required sample size was calculated with the WinEpiscope 2.0 software [61]. Assuming an expected maximal prevalence of 1% for virus positive animals, with a CI of 95% and an accepted error of 5%, we obtained a sample size of 96 (population size [PS] = 100) to 299 (PS = ∞) animals for detection of disease. For prevalence estimation (95% CI, 5% accepted error), sample size ranged from 14–80 (for an expected prevalence [EP] = 1-50%, PS = 100) to 16–385 (EP = 1-50%, PS = ∞) animals. Compromising the needs for a reliable epidemiological study and budget restrictions, we aimed at 300 animals per species for the whole country (i.e., total of 1’200 animals), distributed among sampling units considering hunting statistics [55]. In order to evaluate a possible time-trend, we attempted to reach this sample size for two consecutive years.

### Sample collection and animals

Sampling kits included sterile tubes, a syringe and gloves for blood collection, a questionnaire to record biological information on the sampled animal and an information sheet for age estimation on the basis of tooth replacement [62]. Kits were sent to the hunting authorities for further distribution to the competent local game wardens or hunters, who collected blood samples from hunted game. Additionally, few animals found dead or shot due to suspected disease and submitted for necropsy to the Centre for Fish and Wildlife Health (FIWI) or captured in the fields in the frame of several wildlife projects, were also sampled and included in the study. Capture and sampling of living animals (n = 24) were carried out by experienced personal, following routine protocols and with the authorizations of the competent authorities, as requested by the Swiss legislation. In hunted and necropsied animals, blood was taken either directly from the heart or from the body cavities. In living animals, blood was drawn from the jugular vein under anesthesia. Blood samples were transferred into tubes with and without anticoagulant (EDTA) and sent to the laboratory. Samples for serum were centrifuged immediately upon reception. Aliquots of sera and whole blood were stored at −20°C until analysis.

Blood samples of 1’877 wild ruminants were collected all over the country (Figure 1A). Slightly more males (n = 997) than females (n = 872) were sampled, while no information was available for eight animals. Individuals were classified in three age categories based on morphological characteristics including body size, tooth wear, and horn growth [62]: kid/fawn (< 1 year), yearling (1 to < 2 years) and adult (≥ 2 years). The majority of the samples came from adult individuals (n = 1349), followed by yearlings (n = 300) and fawns/kids (n = 219). Information on age was not available for nine animals. All samples were tested for antibodies, and additionally for the presence of viral RNA if available blood quantities were sufficient.

### Laboratory analyses

For detection of antibodies against BVDV, serum samples were first screened with an in-house ELISA [63] and positive or indeterminate samples were additionally tested by a Serum Neutralization Test (SNT) as described previously [64]. Briefly, 100 TCID50 (50% tissue culture infective dose) of BVDV strain R1935/72, [GenBank: U96333.1] were added to a two fold dilution series (1:4 to 1:512) of heat inactivated sample sera (56°C for 30 min) diluted in Earles-MEM (Earle’s minimal essential medium, Flow Laboratories, Allschwil, Switzerland) and incubated for 60 min at 37°C and 5% CO2. Each dilution was added to four wells of microtitre trays (TPP, Trasadingen, Switzerland) containing a sub-confluent (80%) monolayer of foetal bovine turbinate cells. Microtitre trays were subsequently incubated for 5 days at 37°C and 5% CO2 and finally scored for a cytopathic effect (cp BVDV-1).

Titres were calculated according to Spearman-Kaerber [65,66]. According to preliminary study titre values equal to or greater than 1:8 were considered positive [57]. For detection of BVDV RNA, all seronegative samples were tested with real-time RT-PCR in pools of 10, while ELISA positive samples were tested individually. RNA extraction was performed using the Qiagen BioRobot Universal Platform (Qiagen Instruments AG, Hombrechtikon, Switzerland) with the QIAamp Virus BioRobot MDx Kit (Qiagen AG, Hombrechtikon, Switzerland) according to the manufacturer’s instructions. The subsequent BVDV real-time RT-PCR was done with the cador BVDV RT-PCR Kit (Qiagen AG) according to the manufacturer’s instructions but with the following modifications: Only 20μl final volume was used (12μl master mix + 8μl sample) and the run was performed in an Applied Biosystems 7500 Real-Time PCR System (Life Technologies Europe, CH-6300 Zug, Switzerland). The RT-PCR reaction of the BVDV real-time positive sample was done with the QIAGEN OneStep RT-PCR Kit (Qiagen AG) according to the manufacturer’s instructions and with the pan-pesti primer pair 324/326 [67].

PCR products were sent to Microsynth AG, Balgach, Switzerland for sequencing. For sequence analysis and phylogenetic tree, the alignment was conducted with the program MUSCLE [68]. For evolutionary analysis, the program MEGA5 [69] was used. The evolutionary history was inferred using the Neighbor-Joining method [70]. The evolutionary distances were computed using the Maximum Composite Likelihood method [71].

### Questionnaire survey on interspecific interactions

The occurrence and type of interactions between wild and domestic ruminant species in an alpine environment were documented with a questionnaire survey among game wardens in a selected area in the Swiss Alps. Participants to the survey were professional game wardens, i.e., field specialists having good knowledge on local wildlife. With the support of the cantonal hunting office, blank questionnaires were sent by e-mail to all participants, and filled forms were mailed to the first author. Whenever requested, the latter subsequently gained additional information or clarifications on filled questionnaires through telephone interviews to increase the reliability and validity of the survey.

The questionnaire survey was carried out in the sampling unit South-East, which corresponds to the canton of Graubünden (7,105 km^2^; Figure 1). This canton was selected for several reasons: 1) all four indigenous wild ruminant species (roe deer, red deer, chamois and ibex) and domestic ruminants (cattle, sheep and goat) are widespread, 2) there are numerous alpine summer pastures traditionally used for livestock during the summer grazing period, and 3) an efficient network of game wardens covers the whole cantonal territory. Furthermore, the canton borders three countries (Austria, Italy and the Principality of Liechtenstein) and therefore corresponds to a geographical area of major importance for the surveillance of diseases that may spread from neighboring countries [11,72].

The canton of Graubünden is characterized by a mountainous landscape (altitude range: 260-4’049 m a.s.l.), with a mean altitude of 2,100 m a.s.l. Forty percent of the surface is considered as unproductive vegetation, 30% as forest and the rest as residential/agricultural area. Human density (27 people per square kilometer) is the lowest of the country, and 40% of the human population lives above 1’000 m a.s.l. There are about 700 alpine pastures in the whole canton, which equals 1,689 km^2^ (approximately 24% of the total cantonal surface, i.e. highest proportion of alpine pastures of the country). For wildlife management purposes, the canton is divided in 60 surveillance sectors organized in 12 districts. Each sector is under the responsibility of a professional full- or part-time game warden, with a full-time game warden leading each district. Each game warden covers the same sub-district throughout his duty period.

Questionnaires included four sub-topics and were mailed in two steps. In July of 2010, a first questionnaire gathered information on 1) proximity between species: physical contact; encounter of less than 50 m (< 50 m) or more than 50 m (> 50 m); non-simultaneous occupation of the same area; 2) duration of encounters (record of encounters at < 50 m for more than 1 h); and 3) type of interactions observed: mixing of herds when grazing; use of the same natural feeding resources; same salt lick; or same resting places. In October 2011 a second, shorter questionnaire focused on 4) use of the same anthropogenic food sources (wildlife supplemental feeding, livestock food, and other food sources on private grounds). For data analysis, wildlife supplemental feeding was considered as “intentional” feeding, while livestock food resources were classified as “non-intentional” feeding. For other food sources on private grounds (e.g. dry bread spread for wildlife or garden waste used by wild species), clear differentiation between intentional and non-intentional feeding was not feasible as both were common on the same grounds (“mixed”).

Each questionnaire (in German, available from the corresponding author upon request) consisted of four tables (one for each wild ruminant species) including a list of closed questions and space for personal comments and additions. For each question, multiple choice answers corresponded to the frequency of observation of the listed interaction: 1) never, 2) rarely, i.e., no more than once per year, 3) more than once per year. The period of observation was defined individually for each questionnaire as the number of duty-years of the responding game warden.

### Data management and statistical analysis

Data handling, validating, cleaning and coding were done in MS Excel^Â©^ spread sheets followed by transfer to the NCSS 2007 software (Hintze, J. (2007). NCSS 2007. NCSS, LLC. Kaysville, Utah, USA. http://www.ncss.com) for statistical analyses. Prevalences were calculated assuming test sensitivity and specificity of 100%. The two-tailed Fischer’s exact test (FET) was used to determine differences in prevalence of infection among age classes, sexes, geographical regions and sampling periods (serological and virological survey). The FET was also applied to assess differences between percentages of observations of interspecific interactions (proximity, duration and type of interaction including use of anthropogenic food resource) for each combination of two different animal species. The level of significance was set at *p* < 0.05. Non-interpretable serological and PCR results were not included in the statistical analyses. Maps were designed using the gvSIG software, version 1.11.0 final (Â© gvSIG Association).

## Competing interests

The authors declare that they have no competing interest.

## Authors’ contributions

JC contributed to sample collection, performed the serological screening tests, coordinated the questionnaire survey, analyzed the data, and drafted the manuscript. HRV contributed to the study design and supervised the serological screening. CH performed the PCR for BVDV. HPS supervised the PCR for BVD and performed the BVD strain comparison. HRV, CH and HPS contributed to the interpretation of laboratory results. MPRD designed and coordinated the study, contributed to sample collection, questionnaire design and data analysis, and drafted the manuscript. All authors critically read and approved the final manuscript.

## Supplementary Material

Additional file 1 **Raw data of the questionnaire survey on interactions among wild caprids (Alpine chamois and ibex) and among cervids (roe and red deer).** Numbers refer to the number of game-wardens having reported the corresponding observation, i.e., number of analysed questionnaires (N total) and reported frequency of observations (1: never observed; 2: observed no more than once per year; 3: observed more than once per year). Click here for file

Additional file 2 **Raw data of the questionnaire survey on interactions between Alpine chamois and cervids.** Numbers refer to the number of game-wardens having reported the corresponding observation, i.e., number of analysed questionnaires (N total) and reported frequency of observations (1: never observed; 2: observed no more than once per year; 3: observed more than once per year). Click here for file

Additional file 3 **Raw data of the questionnaire survey on interactions between Alpine ibex and cervids.** Numbers refer to the number of game-wardens having reported the corresponding observation, i.e., number of analysed questionnaires (N total) and reported frequency of observations (1: never observed; 2: observed no more than once per year; 3: observed more than once per year). Click here for file

Additional file 4 **Raw data of the questionnaire survey on interactions between roe deer and domestic ruminants.** Numbers refer to the number of game-wardens having reported the corresponding observation, i.e., number of analysed questionnaires (N) and reported frequency of observations (1: never observed; 2: observed no more than once per year; 3: observed more than once per year). Click here for file

Additional file 5 **Raw data of the questionnaire survey on interactions between red deer and domestic ruminants.** Numbers refer to the number of game-wardens having reported the corresponding observation, i.e., number of analysed questionnaires (N) and reported frequency of observations (1: never observed; 2: observed no more than once per year; 3: observed more than once per year). Click here for file

Additional file 6 **Raw data of the questionnaire survey on interactions between Alpine chamois and domestic ruminants.** Numbers refer to the number of game-wardens having reported the corresponding observation, i.e., number of analysed questionnaires (N) and reported frequency of observations (1: never observed; 2: observed no more than once per year; 3: observed more than once per year). Click here for file

Additional file 7 **Raw data of the questionnaire survey on interactions between Alpine ibex and domestic ruminants.** Numbers refer to the number of game-wardens having reported the corresponding observation, i.e., number of analysed questionnaires (N) and reported frequency of observations (1: never observed; 2: observed no more than once per year; 3: observed more than once per year). Click here for file
