# Supplementary material for: Bovine viral diarrhea virus in free-ranging wild ruminants in Switzerland: low prevalence of infection despite regular interactions with domestic livestock
Source: BMC Vet Res. 2012 Oct 29;8:204. doi: 10.1186/1746-6148-8-204 (PMC3514304; doi:10.1186/1746-6148-8-204)
Supplement: Additional file 1 — Raw data of the questionnaire survey on interactions among wild caprids (Alpine chamois and ibex) and among cervids (roe and red deer). Numbers refer to the number of game-wardens having reported the corresponding observation, i.e., number of analysed questionnaires (N total) and reported frequency of observations (1: never observed; 2: observed no more than once per year; 3: observed more than once per year). [file 1746-6148-8-204-S1.pdf]

Additional file 1: Interactions among wild caprids (Alpine chamois and ibex) and among cervids (roe and red deer). Numbers refer to the number of game-wardens having reported the corresponding observation, i.e., number of analyzed questionnaires (N total) and reported frequency of observations (1: never observed; 2: observed no more than once per year; 3: observed more than once per year).

**CHAMOIS - IBEX**	**N total**	**Reported frequency of observation**		
		**1**	**2**	**3**
**Proximity between species**				
Physical contact	35	24	6	5
Encounter of less than 50 m	35	0	5	30
Encounter of more than 50 m	35	0	0	35
Non-simultaneous occupation of the same area	35	0	2	33
**Duration of encounters**				
Encounters of less than 50 m for more than 1 h	35	4	8	23
**Type of interactions**				
Mixing of herds when grazing	35	2	5	28
Use of the same natural feeding resources	35	2	0	33
Use of the same same salt lick	35	3	2	30
Use of the same same resting places	35	4	6	25
**Anthropogenic food sources**				
Wildlife supplemental feeding	45	43	2	0
Livestock food sources	45	45	0	0
Other food sources on private grounds	45	45	0	0

**ROE DEER - RED DEER**	**N total**	**Reported frequency of observation**		
		**1**	**2**	**3**
**Proximity between species**				
Physical contact	40	36	4	0
Encounter of less than 50 m	40	0	7	33
Encounter of more than 50 m	40	0	1	39
Non-simultaneous occupation of the same area	40	1	0	39
**Duration of encounters**				
Encounters of less than 50 m for more than 1 h	40	11	17	12
**Type of interactions**				
Mixing of herds when grazing	40	5	15	20
Use of the same natural feeding resources	40	2	7	31
Use of the same same salt lick	40	17	4	19
Use of the same same resting places	40	6	14	20
**Anthropogenic food sources**				
Wildlife supplemental feeding	50	41	0	9
Livestock food sources	50	25	9	16
Other food sources on private grounds	50	18	9	23
