# Supplementary material for: Bovine viral diarrhea virus in free-ranging wild ruminants in Switzerland: low prevalence of infection despite regular interactions with domestic livestock
Source: BMC Vet Res. 2012 Oct 29;8:204. doi: 10.1186/1746-6148-8-204 (PMC3514304; doi:10.1186/1746-6148-8-204)
Supplement: Additional file 2 — Raw data of the questionnaire survey on interactions between Alpine chamois and cervids. Numbers refer to the number of game-wardens having reported the corresponding observation, i.e., number of analysed questionnaires (N total) and reported frequency of observations (1: never observed; 2: observed no more than once per year; 3: observed more than once per year). [file 1746-6148-8-204-S2.pdf]

Additional file 2: Interactions between Alpine chamois and cervids. Numbers refer to the number of game-wardens having reported the corresponding observation, i.e., number of analyzed questionnaires (N total) and reported frequency of observations (1: never observed; 2: observed no more than once per year; 3: observed more than once per year).

**CHAMOIS - ROE DEER**	**N total**	**Reported frequency of observation**		
		**1**	**2**	**3**
**Proximity between species**				
Physical contact	40	40	0	0
Encounter of less than 50 m	40	10	16	14
Encounter of more than 50 m	40	5	12	23
Non-simultaneous occupation of the same area	40	3	10	27
**Duration of encounters**				
Encounters of less than 50 m for more than 1 h	40	23	10	7
**Type of interactions**				
Mixing of herds when grazing	40	15	18	7
Use of the same natural feeding resources	40	6	12	22
Use of the same same salt lick	40	19	10	11
Use of the same same resting places	40	26	9	5
**Anthropogenic food sources**				
Wildlife supplemental feeding	50	49	0	1
Livestock food sources	50	49	1	0
Other food sources on private grounds	50	49	1	0

**CHAMOIS - RED DEER**	**N total**	**Reported frequency of observation**		
		**1**	**2**	**3**
**Proximity between species**				
Physical contact	40	37	2	1
Encounter of less than 50 m	40	2	15	23
Encounter of more than 50 m	40	0	7	33
Non-simultaneous occupation of the same area	40	2	8	30
**Duration of encounters**				
Encounters of less than 50 m for more than 1 h	40	16	11	13
**Type of interactions**				
Mixing of herds when grazing	40	11	14	15
Use of the same natural feeding resources	40	3	8	29
Use of the same same salt lick	40	8	11	21
Use of the same same resting places	40	13	11	16
**Anthropogenic food sources**				
Wildlife supplemental feeding	50	48	1	1
Livestock food sources	50	50	0	0
Other food sources on private grounds	50	48	2	0
