# Supplementary material for: Bovine viral diarrhea virus in free-ranging wild ruminants in Switzerland: low prevalence of infection despite regular interactions with domestic livestock
Source: BMC Vet Res. 2012 Oct 29;8:204. doi: 10.1186/1746-6148-8-204 (PMC3514304; doi:10.1186/1746-6148-8-204)
Supplement: Additional file 3 — Raw data of the questionnaire survey on interactions between Alpine ibex and cervids. Numbers refer to the number of game-wardens having reported the corresponding observation, i.e., number of analysed questionnaires (N total) and reported frequency of observations (1: never observed; 2: observed no more than once per year; 3: observed more than once per year). [file 1746-6148-8-204-S3.pdf]

Additional file 3: Interactions between Alpine ibex and cervids. Numbers refer to the number of game-wardens having reported the corresponding observation, i.e., number of analyzed questionnaires (N total) and reported frequency of observations (1: never observed; 2: observed no more than once per year; 3: observed more than once per year).

**IBEX - ROE DEER**	**N total**	**Reported frequency of observation**		
		**1**	**2**	**3**
**Proximity between species**				
Physical contact	35	35	0	0
Encounter of less than 50 m	35	24	8	3
Encounter of more than 50 m	35	21	7	7
Non-simultaneous occupation of the same area	35	18	8	9
**Duration of encounters**				
Encounters of less than 50 m for more than 1 h	35	28	7	0
**Type of interactions**				
Mixing of herds when grazing	35	30	4	1
Use of the same natural feeding resources	35	19	14	2
Use of the same same salt lick	35	28	7	0
Use of the same same resting places	35	33	1	1
**Anthropogenic food sources**				
Wildlife supplemental feeding	45	45	0	0
Livestock food sources	45	45	0	0
Other food sources on private grounds	45	45	0	0

**IBEX - RED DEER**	**N total**	**Reported frequency of observation**		
		**1**	**2**	**3**
**Proximity between species**				
Physical contact	35	35	0	0
Encounter of less than 50 m	35	17	14	4
Encounter of more than 50 m	35	12	11	12
Non-simultaneous occupation of the same area	35	12	16	7
**Duration of encounters**				
Encounters of less than 50 m for more than 1 h	35	28	5	2
**Type of interactions**				
Mixing of herds when grazing	35	26	7	2
Use of the same natural feeding resources	35	10	10	15
Use of the same same salt lick	35	18	10	7
Use of the same same resting places	35	26	6	3
**Anthropogenic food sources**				
Wildlife supplemental feeding	45	45	0	0
Livestock food sources	45	45	0	0
Other food sources on private grounds	45	45	0	0
