# Supplementary material for: Bovine viral diarrhea virus in free-ranging wild ruminants in Switzerland: low prevalence of infection despite regular interactions with domestic livestock
Source: BMC Vet Res. 2012 Oct 29;8:204. doi: 10.1186/1746-6148-8-204 (PMC3514304; doi:10.1186/1746-6148-8-204)
Supplement: Additional file 4 — Raw data of the questionnaire survey on interactions between roe deer and domestic ruminants. Numbers refer to the number of game-wardens having reported the corresponding observation, i.e., number of analysed questionnaires (N) and reported frequency of observations (1: never observed; 2: observed no more than once per year; 3: observed more than once per year). [file 1746-6148-8-204-S4.pdf]

Additional file 4: Interactions between roe deer and domestic ruminants. Numbers refer to the number of game-wardens having reported the corresponding observation, i.e., number of analyzed questionnaires (N) and reported frequency of observations (1: never observed; 2: observed no more than once per year; 3: observed more than once per year).

**ROE DEER with**	**CATTLE**				**SHEEP**				**GOAT**			
	**N**	**1**	**2**	**3**	**N**	**1**	**2**	**3**	**N**	**1**	**2**	**3**
**Proximity between species**												
Physical contact	40	39	1	0	38	37	1	0	37	37	0	0
Encounter of less than 50 m	40	6	9	25	38	11	13	14	37	13	14	10
Encounter of more than 50 m	40	3	5	32	38	8	7	23	37	10	10	17
Non-simultaneous occupation of the same area	40	3	0	37	38	3	7	28	37	6	6	25
**Duration of encounters**												
Encounters of less than 50 m for more than 1 h	40	16	16	8	38	26	11	1	37	25	12	0
**Type of interactions**												
Mixing of herds when grazing	40	13	10	17	38	23	11	4	37	25	10	2
Use of the same natural feeding resources	40	6	7	27	38	9	7	22	37	10	12	15
Use of the same same salt lick	40	22	7	11	38	21	9	8	37	23	9	5
Use of the same same resting places	40	21	11	8	38	27	7	4	37	28	6	3
**Anthropogenic food sources**												
Wildlife supplemental feeding	50	49	1	0	49	48	1	0	48	47	1	0
Livestock food sources	50	31	8	11	49	40	6	3	48	42	4	2
Other food sources on private grounds	50	44	5	1	49	47	2	0	48	47	1	0
