# Supplementary material for: Bovine viral diarrhea virus in free-ranging wild ruminants in Switzerland: low prevalence of infection despite regular interactions with domestic livestock
Source: BMC Vet Res. 2012 Oct 29;8:204. doi: 10.1186/1746-6148-8-204 (PMC3514304; doi:10.1186/1746-6148-8-204)
Supplement: Additional file 5 — Raw data of the questionnaire survey on interactions between red deer and domestic ruminants. Numbers refer to the number of game-wardens having reported the corresponding observation, i.e., number of analysed questionnaires (N) and reported frequency of observations (1: never observed; 2: observed no more than once per year; 3: observed more than once per year). [file 1746-6148-8-204-S5.pdf]

Additional file 5: Interactions between red deer and domestic ruminants. Numbers refer to the number of game-wardens having reported the corresponding observation, i.e., number of analyzed questionnaires (N) and reported frequency of observations (1: never observed; 2: observed no more than once per year; 3: observed more than once per year).

**RED DEER with**		**CATTLE**				**SHEEP**				**GOAT**	
	**N**	**1**	**2**	**3**	**N**	**1**	**2**	**3**	**N**	**1**	**2**	**3**
**Proximity between species**												
Physical contact	40	37	1	2	38	38	0	0	37	37	0	0
Encounter of less than 50 m	40	4	15	21	38	12	18	8	37	14	16	7
Encounter of more than 50 m	40	3	6	31	38	6	12	20	37	9	13	15
Non-simultaneous occupation of the same area	40	1	6	33	38	7	9	22	37	10	11	16
**Duration of encounters**												
Encounters of less than 50 m for more than 1 h	40	17	11	12	38	25	10	3	37	26	9	2
**Type of interactions**												
Mixing of herds when grazing	40	12	16	12	38	21	10	7	37	25	10	2
Use of the same natural feeding resources	40	2	4	34	38	6	9	23	37	10	11	16
Use of the same same salt lick	40	14	12	14	38	18	6	14	37	22	10	5
Use of the same same resting places	40	16	7	17	38	21	9	8	37	26	6	5
**Anthropogenic food sources**												
Wildlife supplemental feeding	50	47	0	3	49	49	0	0	48	48	0	0
Livestock food sources	50	19	4	27	49	30	9	10	48	36	4	8
Other food sources on private grounds	50	44	2	4	49	41	5	3	48	47	1	0
