# Supplementary material for: Bovine viral diarrhea virus in free-ranging wild ruminants in Switzerland: low prevalence of infection despite regular interactions with domestic livestock
Source: BMC Vet Res. 2012 Oct 29;8:204. doi: 10.1186/1746-6148-8-204 (PMC3514304; doi:10.1186/1746-6148-8-204)
Supplement: Additional file 6 — Raw data of the questionnaire survey on interactions between Alpine chamois and domestic ruminants. Numbers refer to the number of game-wardens having reported the corresponding observation, i.e., number of analysed questionnaires (N) and reported frequency of observations (1: never observed; 2: observed no more than once per year; 3: observed more than once per year). [file 1746-6148-8-204-S6.pdf]

Additional file 6: Interactions between Alpine chamois and domestic ruminants. Numbers refer to the number of game-wardens having reported the corresponding observation, i.e., number of analyzed questionnaires (N) and reported frequency of observations (1: never observed; 2: observed no more than once per year; 3: observed more than once per year).

**CHAMOIS with**	**CATTLE**				**SHEEP**				**GOAT**			
	**N**	**1**	**2**	**3**	**N**	**1**	**2**	**3**	**N**	**1**	**2**	**3**
**Proximity between species**												
Physical contact	40	40	0	0	38	37	1	0	37	33	3	1
Encounter of less than 50 m	40	10	17	13	38	6	13	19	37	10	12	15
Encounter of more than 50 m	40	4	9	27	38	3	4	31	37	6	9	22
Non-simultaneous occupation of the same area	40	7	7	26	38	2	2	34	37	7	6	24
**Duration of encounters**												
Encounters of less than 50 m for more than 1 h	40	21	10	9	38	14	12	12	37	23	6	8
**Type of interactions**												
Mixing of herds when grazing	40	22	7	11	38	19	7	12	37	23	8	6
Use of the same natural feeding resources	40	6	11	23	38	4	2	32	37	6	7	24
Use of the same same salt lick	40	13	11	16	38	9	6	23	37	14	6	17
Use of the same same resting places	40	23	13	4	38	12	8	18	37	15	11	11
**Anthropogenic food sources**												
Wildlife supplemental feeding	50	50	0	0	49	49	0	0	48	48	0	0
Livestock food sources	50	48	2	0	49	45	3	1	48	46	2	0
Other food sources on private grounds	50	48	1	1	49	47	2	0	48	47	1	0
