# Supplementary material for: Bovine viral diarrhea virus in free-ranging wild ruminants in Switzerland: low prevalence of infection despite regular interactions with domestic livestock
Source: BMC Vet Res. 2012 Oct 29;8:204. doi: 10.1186/1746-6148-8-204 (PMC3514304; doi:10.1186/1746-6148-8-204)
Supplement: Additional file 7 — Raw data of the questionnaire survey on interactions between Alpine ibex and domestic ruminants. Numbers refer to the number of game-wardens having reported the corresponding observation, i.e., number of analysed questionnaires (N) and reported frequency of observations (1: never observed; 2: observed no more than once per year; 3: observed more than once per year). [file 1746-6148-8-204-S7.pdf]

Additional file 7: Interactions between Alpine ibex and domestic ruminants. Numbers refer to the number of game-wardens having reported the corresponding observation, i.e., number of analyzed questionnaires (N) and reported frequency of observations (1: never observed; 2: observed no more than once per year; 3: observed more than once per year).

**IBEX with**	**CATTLE**				**SHEEP**				**GOAT**			
	**N**	**1**	**2**	**3**	**N**	**1**	**2**	**3**	**N**	**1**	**2**	**3**
**Proximity between species**												
Physical contact	35	35	0	0	33	28	4	1	32	27	3	2
Encounter of less than 50 m	35	21	10	4	33	9	7	17	32	11	9	12
Encounter of more than 50 m	35	16	10	9	33	5	4	24	32	9	5	18
Non-simultaneous occupation of the same area	35	12	11	12	33	4	5	24	32	10	4	18
**Duration of encounters**												
Encounters of less than 50 m for more than 1 h	35	31	4	0	33	16	11	6	32	18	9	5
**Type of interactions**												
Mixing of herds when grazing	35	29	5	1	33	15	9	9	32	17	11	4
Use of the same natural feeding resources	35	14	13	8	33	4	4	25	32	9	4	19
Use of the same same salt lick	35	23	8	4	33	8	5	20	32	13	5	14
Use of the same same resting places	35	30	4	1	33	12	10	11	32	16	5	11
**Anthropogenic food sources**												
Wildlife supplemental feeding	45	45	0	0	44	43	1	0	43	43	0	0
Livestock food sources	45	44	1	0	44	43	1	0	43	42	1	0
Other food sources on private grounds	45	45	0	0	44	44	0	0	43	43	0	0
